# An optimal condition for the evaluation of human brown adipose tissue by infrared thermography

**DOI:** 10.1371/journal.pone.0220574

**Published:** 2019-08-26

**Authors:** Shinsuke Nirengi, Hitoshi Wakabayashi, Mami Matsushita, Masayuki Domichi, Shinichi Suzuki, Shin Sukino, Akiko Suganuma, Yaeko Kawaguchi, Takeshi Hashimoto, Masayuki Saito, Naoki Sakane

**Affiliations:** 1 Division of Preventive Medicine, Clinical Research Institute, National Hospital Organization Kyoto Medical Center, Kyoto, Japan; 2 Laboratory of Environmental Ergonomics, Faculty of Engineering, Hokkaido University, Sapporo, Japan; 3 Tenshi College, Department of Nutrition, Sapporo, Japan; 4 D-eyes Inc., Osaka, Japan; 5 Hokkaido University, Sapporo, Japan; St. Joseph's Hospital and Medical Center, UNITED STATES

## Abstract

Brown adipose tissue (BAT) is responsible for non-shivering thermogenesis and is an attractive therapeutic target for combating obesity and related diseases. Human BAT activity has been evaluated by ^18^F-fluorodeoxyglucose-positron emission tomography/computed tomography (^18^FDG-PET/CT) under acute cold exposure, but the method has some serious limitations, including radiation exposure. Infrared thermography (IRT) may be a simple and less-invasive alternative to evaluate BAT activity. In the present study, to establish an optimal condition for IRT, using a thermal imaging camera, skin temperature was measured in the supraclavicular region close to BAT depots (T_scv_) and the control chest region (T_c_) in 24 young healthy volunteers. Their BAT activity was assessed as the maximal standardized uptake value (SUV_max_) by ^18^FDG-PET/CT. Under a warm condition at 24–27°C, no significant correlation was found between the IRT parameters (T_scv_, T_c,_, and the difference between T_scv_ and T_c,_, Δtemp) and SUV_max_, but 30–120 min after cold exposure at 19°C, T_scv_ and Δtemp were significantly correlated with SUV_max_ (*r* = 0.40–0.48 and *r* = 0.68–0.76). Δtemp after cold exposure was not affected by mean body temperature, body fatness, and skin blood flow. A lower correlation (*r* = 0.43) of Δtemp with SUV_max_ was also obtained when the participant’s hands were immersed in water at 18°C for 5 min. Receiver operating characteristic analysis revealed that Δtemp after 30–60 min cold exposure can be used as an index for BAT evaluation with 74% sensitivity, 92% specificity, and 79% diagnostic accuracy. Thus, IRT may be useful as a simple and less-invasive method for evaluating BAT, particularly for large-scale screening and longitudinal repeat studies.

## Introduction

Brown adipose tissue (BAT) is responsible for non-shivering thermogenesis (NST) and is therefore involved in the regulation of whole-body energy expenditure and body fatness [1]. In humans, the current gold standard method to assess BAT is ^18^F-fluorodeoxyglucose (FDG)–positron emission tomography (PET) in combination with computed tomography (CT) and cold exposure, which uses cold-activated glucose uptake as an index of BAT activity [2]. However, this ^18^FDG-PET/CT method has some serious drawbacks such as radiation exposure, the need for cold exposure, and the high cost of the device, which have limited its frequent use in both experimental and clinical studies. Although several alternative methods to overcome these limitations have been developed, including magnetic resonance imaging [3], near-infrared time-resolved spectroscopy [4], and contrast-enhanced ultrasound [5], they are also relatively expensive and not yet soundly confirmed for their validity and reliability [6].

There have been reports to assess the thermogenic activity of BAT by monitoring the temperature of the skin (T_sk_) overlying BAT depots. A few studies using a wire-less thermistor probe revealed that cold-induced changes in T_sk_ of the supraclavicular region (T_scv_) close to BAT depots positively correlated with the activity and volume of BAT estimated by ^18^FDG-PET/CT [7,8]. Infrared thermography (IRT) can also be used to evaluate T_sk_ with visualization by measuring the infrared radiation emitted from the body surface. Jang et al. showed by IRT that differences between T_sk_ in a control chest region and T_scv_ (Δtemp) were greater in subjects having higher BAT activities after a 2-h cold exposure [9]. Furthermore, a significant relationship between the IRT method and ^18^FDG-PET/CT also has been confirmed [9,10]. However, during the cold exposure experiments of these studies, T_sk_ was measured before and after a 2-h cold exposure protocol, where the subjects with light-clothing were kept in a room at 19°C (cold exposure) or on mattresses perfused with cooled water at ~17°C; these protocols may not induce muscle shivering, but are apparently uncomfortable, stressful, and intolerable for most individuals, particularly those with cardiovascular diseases. Therefore, less invasive and easier protocols are needed for frequent assessment of BAT, in both experimental and clinical studies.

Symonds et al. [11] and Ang et al. [12] tested the feasibility of IRT as a non-invasive method by monitoring the changes in T_scv_ 5 min after placing the hand and/or feet of the participant in water at 20°C. This hand immersion protocol is apparently much less invasive and is easily applicable in various experimental and clinical settings; however, they did not validate the correlation of their data with the BAT activity assessed by ^18^FDG-PET/CT. In the present study, to establish optimal conditions for IRT assessment of human BAT, we monitored the response of T_sk_ to cold exposure for 10–120 min, in healthy subjects with a wide range of BAT activity. We also examined T_sk_ response after 5-min hand immersion in the same subjects, and compared the two protocols. Our results revealed that Δtemp, only after 30-min exposure to cold at 19°C, correlated well with the BAT activity assessed by ^18^FDG-PET/CT, indicating that this protocol can be used for BAT evaluation with an accuracy of approximately 80%.

## Methods

Twenty-four healthy male volunteers (age: 23.5 ± 3.6 years; body mass index [BMI]: 21.6 ± 2.5 kg/m^2^) participated in this study in winter from December 2017 to March 2018. This study was carried out in accordance with the principles of the Declaration of Helsinki (Fortaleza 2013). The protocol was approved by the institutional review boards of Kyoto Medical Center (no. 15–092) and was registered at the University Hospital Medical Information Network (UMIN) center (UMIN000029206). Written informed consent was obtained from all participants.

### ^18^FDG-PET/CT

After overnight fasting for ~12 h, subjects were exposed to cold by being kept in an air-conditioned room at 19°C with standardized light clothing (a patient gown), with intermittent placement of their feet on an ice block wrapped in cloth for ~4 min at 5-min intervals to avoid cooling-associated pain [13]. After 1 h under these cold conditions, each subject was intravenously injected with ^18^F-FDG (1.66–5.18 mega [106] Becquerel (MBq)/kg body weight) and kept under the same cold conditions. At 1 h after the ^18^F-FDG injection, ^18^FDG-PET/CT scans were obtained with a PET/CT system (Aquiduo; Toshiba Medical Systems, Otawara, Japan). BAT activity in both the right and left supraclavicular regions was quantified based on the maximum standardized uptake value (SUV_max_), defined as the radioactivity per ml within the region of interest divided by the injected dose in mBq/g body weight. BAT was defined as tissue with Hounsfield units −300 to −10 on CT with an SUV ≥ 1.5. PET and CT images were co-registered and analyzed using VOXBASE workstation (J-MAC System, Sapporo, Japan).

### IRT

IRT was carried out using a thermal imaging camera (DE-TC1000T; D-eyes Inc., Osaka, Japan) fastened to a tripod. The thermal resolution was 160 × 120. The T_scv_ of both the right and left sides was measured from each image. The T_sk_ of the chest region (T_c_) immediately lateral to the sternum approximating the second intercostal space, which is apart from the underlying BAT depots, was simultaneously measured as a control [13]. The subjects fasted for ~12 h, wore a light patient gown (about 0.2 clo), and underwent IRT successively for the following two tests: 5-min hand immersion into 18°C water and 120-min cold exposure at 19°C, as described below. IRT images were analyzed using a modified (D-eyes Inc.) version of ThermalCam v.1.1.0.9 software (Laon People Inc., Seoul, Korea).

### Cold exposure test

Cold exposure was performed using two adjacently located rooms controlled at 27°C and 19°C, respectively, with 40% relative humidity. The coefficient of variance (CV) was 2.5% in the 27°C room and 1.1% in the 19°C room. Subjects were seated in an upright position looking straight ahead for ~30 min in the 27°C room and underwent IRT and other measurements including skin blood flow (SkBF), then moved to the 19°C room and underwent IRT at 10–30 min intervals for 120 min.

### Hand immersion test

For the hand immersion test, the ambient room temperature and water temperature were 24°C and 18°C, respectively, with 40% relative humidity. The water temperature in a tank was maintained using a thermostatic water circulator (LV-200; Toyo Roshi Kaisha, Tokyo, Japan), and the CV of water temperature was 5.4%. After more than 30 min of rest, the subjects immersed both hands into the water tank for 5 min [11,12].

### Anthropometric parameters and others

BMI was calculated as body weight in kilograms divided by the square of the height in meters, and body fat mass was estimated by the multifrequency bioelectric impedance method (Karada Scan HBF-701; Omron, Kyoto, Japan). Visceral and subcutaneous fat areas at the abdominal level of L4–L5 were estimated from the CT images. Total abdominal fat area was calculated as the sum of visceral and subcutaneous fat areas.

Tympanic and sublingual temperature was measured using an earphone type infrared tympanic thermometer (CET-101; Nipro, Osaka, Japan) and an electronic thermometer before and after 2-h cold exposure (MC-172L; Omron Healthcare Co., Kyoto, Japan), respectively. A small disc-type temperature data logger (Thermochron SL; KN Laboratories, Osaka, Japan) was used to monitor T_sk_ on the forehead, left upper chest, non-dominant ventral forearm, non-dominant ventral middle finger, left shin, and left instep as reported previously [8]. The mean T_sk_ was calculated according to a modified Hardy and DuBois’s equation [14].

SkBF in the supraclavicular region and back (left scapula) was measured using a laser tissue blood flowmeter (FLO-N1; Omegawave, Inc., Tokyo, Japan). Data were sampled using an A/D converter and recorded at 1-s intervals using a personal computer. In the subsequent analysis, artifacts observed in the raw data were eliminated using a 10-s median filter [15]. Before and after 2-h cold exposure, subjects were asked to rate shivering according to a modified version of a previously used scale [16] consisting of four levels: 1 = no shivering, 2 = slight shivering, 3 = moderate shivering, and 4 = heavy shivering. Cold sensation [17] and discomfort [18] were also assessed before and after 2-h cold exposure.

### Statistical analyses

Data are expressed as mean ± standard deviation. Two-way analysis of variance with repeated measures was used to test interactions (group × time) and main effects (group, time). If there was a significant interaction or main effect, time or group differences in variables between baseline and after the test, were analyzed with the paired and unpaired t tests, respectively. The relationship between the data of IRT and ^18^FDG-PET/CT was analyzed by Pearson’s correlation analysis, where SUV_max_ was log-transformed because of the non-normal distribution determined with the Shapiro-Wilk test. Values were considered statistically significant at *P* < 0.05. Receiver operating characteristic (ROC) analysis was performed to evaluate the area under the ROC curve (AUC), sensitivity, specificity, and the accuracy of IRT parameters. Then the AUC of after cold exposure was compared to that of 27°C. The statistical analyses were performed using SPSS v.19 (IBM, Armonk, NY, USA) and Easy R software (Saitama Medical Center, Jichi Medical University, Saitama, Japan) [19].

## Results

^18^FDG-PET/CT revealed that 5 of 24 subjects showed undetectably low BAT activity (SUV_max_ < 1.5), and thus, were defined as BAT-negative, whereas the remaining 19 subjects showed a detectable activity (SUV_max_ = 1.8~26.8), and thus, were defined as BAT-positive. There was no significant difference in the anthropometric parameters between the two subject groups (Table 1).

**Table 1:** Characteristics of study subjects.

Measurement	All	BAT-positive	BAT-negative	*P*-value
Number	24	19	5	-
Age, years	23.5 ± 3.6	23.8 ± 3.8	22.4 ± 2.2	0.44
Height, cm	172.1 ± 4.6	172.6 ± 5.0	170.2 ± 1.7	0.53
Weight, kg	64.0 ± 8.6	65.4 ± 8.9	58.9 ± 5.6	0.24
BMI, kg/m^2^	21.6 ± 2.5	21.9 ± 2.6	20.3 ± 1.9	0.29
Body fat, %	16.6 ± 4.4	17.4 ± 4.4	13.7 ± 3.4	0.22
Skeletal muscle, kg	35.6 ± 1.8	35.3 ± 1.8	36.6 ± 1.5	0.29
Visceral fat area, cm^2^	42.8 ± 31.6	45.0 ± 33.9	34.6 ± 21.3	0.47
Subcutaneous fat area, cm^2^	88.7 ± 54.4	94.3 ± 56.4	67.4 ± 44.9	0.34
Total abdominal fat area, cm^2^	131.5 ± 72.7	139.3 ± 74.5	102.0 ± 63.5	0.31
SUV_max_	7.0 ± 6.9	8.1 ± 6.1	0.6 ± 0.2	< 0.01

Values represent mean ± standard deviation. BAT, brown adipose tissue; BMI, body mass index; SUV_max_, maximal standardized uptake value.

**Fig 1:**
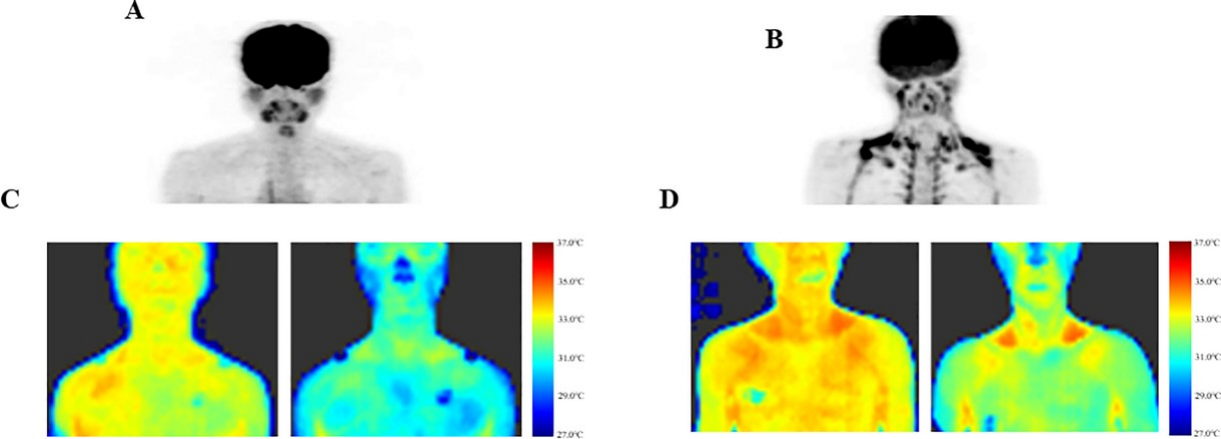
Typical images of ^18^FDG-PET/CT and IRT. Typical images of ^18^FDG-PET/CT in BAT-negative (**A**) and positive subjects (**B**). Typical images of IRT method in BAT-negative (**C**) and positive subjects (**D**) before (left) and after 2-h cold exposure (right).

**Fig 2:**
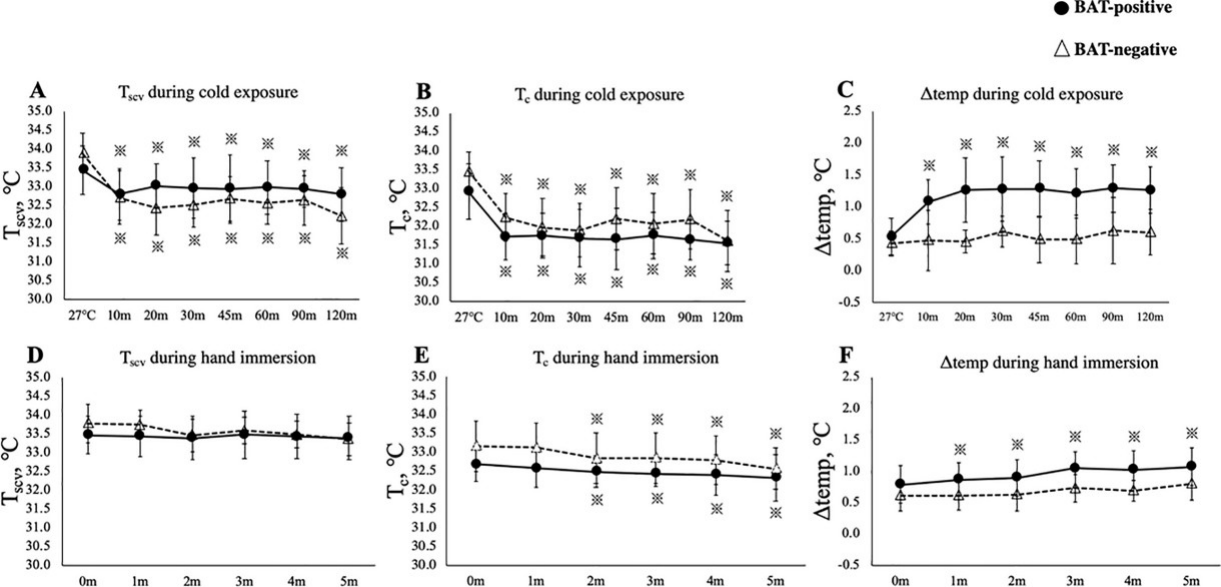
Skin temperature changes after cold exposure and hand immersion. T_scv_, skin temperature of the supraclavicular region; T_c_, skin temperature of the chest region; Δtemp, differences between T_scv_ and T_c_. T_scv_ (**A**), T_c_ (**B**), and Δtemp (**C**) during cold exposure. T_scv_ (**D**), T_c_ (**E**), and Δtemp (**F**) during hand immersion. * vs 27°C or 0 m.

Fig 1 shows typical images of IRT and ^18^FDG-PET/CT at 27°C and 2 h after cold exposure. T_sk_ was considerably different between the two subjects, both under warm (27°C) and cold (19°C) conditions. Despite the individual differences, as summarized in Fig 2A and 2B, under the warm condition at 27°C, T_scv_ was insignificantly higher than T_c_ in both BAT-negative and positive groups. After cold exposure, T_scv_ and T_c_ seemed slightly higher and lower, respectively, in the BAT-positive group. Although neither T_c_ nor T_scv_ was significantly different between the two groups at any time point during the 2-h cold exposure, the cold-induced drop in T_scv_ was significantly smaller (*P* < 0.05) in the BAT-positive group (0.7 ± 0.6°C) than in the BAT-negative group (1.7 ± 1.1°C), while the drop in T_c_ was comparable in the two groups (1.4 ± 0.7°C vs. 1.8 ± 1.0°C; *P* = 0.16).

**Fig 3:**
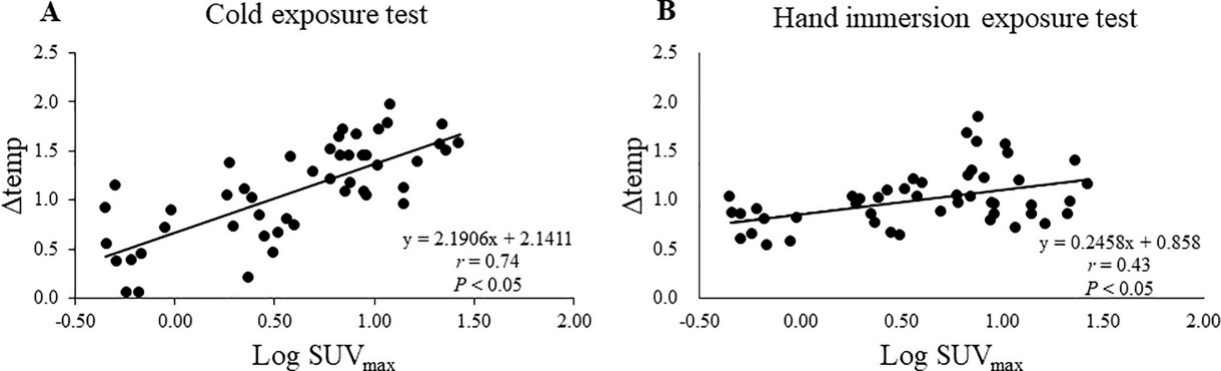
**Relationship between log SUV**_**max**_ **and** Δtemp **in the cold exposure test (A) and the hand immersion test (B).** Δtemp, difference between skin temperature on the supraclavicular region (T_scv_) and that on the chest region (T_c_); SUV_max_, maximal standardized uptake value. The correlation coefficient in the cold exposure test (*r* = 0.74) was significantly higher than that in the hand immersion test (*r* = 0.42) (*P* < 0.05). Data were obtained from both the right and left sides in 24 subjects.

To confirm the effect specific to the supraclavicular region, the difference between T_scv_ and T_c_ was calculated and expressed as Δtemp. As shown in Fig 2C, Δtemp in the BAT-positive group was 0.5 ± 0.3°C at 27°C, rose remarkably and significantly 10 min after cold exposure, reached a steady level of 1.3 ± 0.5°C at 30 min, and was maintained at high levels of 1.2~1.3°C thereafter. In contrast, Δtemp in the BAT-negative group showed no significant change after cold exposure, being 0.5~0.6°C, which was significantly lower (*P* < 0.05) than that of the BAT-positive group. Thus, cold-induced change in Δtemp was observed only in the BAT-positive group. As the supraclavicular region, but not the control chest region, is close to the underlying BAT depots, Δtemp after cold exposure is likely to reflect the thermogenic activity of BAT and would serve as a BAT-specific index. Consistent with this idea, a fairly positive correlation (*r* = 0.74) was observed between the Δtemp at 2-h cold exposure and the BAT activity expressed as log SUV_max_ (Fig 3). Significant correlations with SUV_max_ were also found in T_scv_ itself and the cold-induced T_scv_ change (T_scv_-time), but with lower correlation coefficients (*r* = 0.48 and *r* = 0.59, Table 2). In contrast, neither T_scv_ nor Δtemp at 27°C correlated with SUV_max_. Comparative positive correlations between IRT parameters and log SUV_max_ were also observed even at 30 min after cold exposure, including those for T_scv_ (*r* = 0.40), Δtemp (*r* = 0.68) and T_scv_-time (*r* = 0.57).

**Table 2:** Correlation coefficients between IRT parameters and SUV_max_ in the cold exposure test.

	27°C	19°C (30 min)	19°C (120 min)
T_scv_	-0.24	0.40*	0.48*
T_c_	-0.25	0.08	-0.002
Δtemp	0.16	0.68*	0.74*
T_scv_-time	-	0.57*	0.59*
T_c_-time	-	-0.14	0.22

SUV, standardized uptake value; T_scv_, skin temperature on the supraclavicular region; T_c_, skin temperature on the chest region; Δtemp, differences between T_scv_ and T_c_; T_scv_-time, difference in T_scv_ between 19°C and 27°C; T_c_-time, difference in T_c_ between 19°C and 27°C.

*P < 0.05 (Pearson’s correlation analysis).

We also examined the effects of 2 h-cold exposure on tympanic and sublingual temperatures and skin temperature in various regions including the forehead, forearm, hand, finger, calf, and foot. Similar to the supraclavicular and chest regions, skin temperature in these regions dropped, showing the mean T_sk_ from 33.1°C ± 0.4°C to 29.7°C ± 0.3°C (*P* < 0.01), but no notable difference was found between the BAT-positive and -negative groups (data not shown). The effects of cold-exposure on SkBF were also examined. After 2 h-cold exposure, SkBF decreased by 15.6% (*P* < 0.05) in the back, whereas it did not change in the supraclavicular region (*P* = 0.51), and no difference was found between the BAT-positive and -negative groups. We also investigated the relationship between possible confounding factors and T_sk_ (Table 3). The % body fat was negatively correlated with T_scv_ and T_c_ at 27°C. The mean T_sk_ was positively correlated with T_scv_ and T_c_ at 27°C and T_scv_ at 19°C. The SkBf was positively correlated with T_c_ at 27°C and T_scv_ and T_c_ at 19°C. However, Δtemp did not correlate with any of the parameters or temperatures (Table 3). There was no perceived shivering either before (0.3 ± 0.5) or after (-0.3 ± 0.5) cold exposure, while cold sensation was -2.1 ± 1.0 (-2 = cool) and discomfort was −1.0 ± 0.8 (−1 = uncomfortable) at the end of cold exposure.

**Table 3:** The correlation coefficient between Δtemp and possible confounding factors.

		27°C	19°C 30 min	19°C 120 min
% Body fat	T_scv_	-0.38*	-0.24	-0.09
	T_c_	-0.39*	-0.35*	-0.26
	Δtemp	0.14	0.15	0.20
Mean T_sk_	T_scv_	0.31*	0.37*	0.40*
	T_c_	0.36*	0.39*	0.39*
	Δtemp	-0.21	-0.02	0.10
SkBf	T_scv_	0.07	-	0.56*
	T_c_	0.34*	-	0.36*
	Δtemp	-0.12	-	0.22

T_sk_, skin temperature; skBf, skin blood flow; T_scv_, T_sk_ on the supraclavicular region; T_c_, T_sk_ on the chest region; T_scv_, differences between T_scv_ and T_c_; Δtemp, changes in T_scv_; ΔT_c_, changes in T_c_. The body fat was used as baseline data. The mean T_sk_ was calculated according to a modified Hardy and DuBois’s equation. The skBf was evaluated in the supraclavicular and control regions at 27°C and 19°C.

*P < 0.05 (Pearson’s or Spearman’s correlation analysis).

We also examined the effects of hand immersion in water for the same subjects participating in the above-described cold exposure test. As shown in Fig 2D and 2E, during 5-min hand immersion, T_c_ significantly decreased (*P* < 0.05), while T_scv_ did not change. The calculated Δtemp was increased after the 5-min hand immersion only in the BAT-positive group (*P* < 0.05). A significant positive correlation between Δtemp and log SUV_max_ was found, but the correlation coefficient (*r* = 0.43) was lower than that after cold exposure (Fig 3, Table 4).

**Table 4:** Correlation coefficients between IRT parameters and SUV_max_ in the hand immersion test.

	Before	After (5 min)
T_scv_	-0.01	0.14
T_c_	-0.12	-0.11
Δtemp	0.27	0.43*
T_scv_-time	-	0.17
T_c_-time	-	0.04

SUV, standardized uptake value; T_scv_, skin temperature on the supraclavicular region; T_c_, skin temperature on the chest region; Δtemp, differences between T_scv_ and T_c_; T_scv_-time, difference in T_scv_ between before and after hand immersion; T_c_-time, difference in T_c_ between before and after hand immersion.

*P < 0.05 (Pearson’s correlation analysis).

The ROC analysis between Δtemp and log SUV_max_ revealed that AUCs were 0.80 and 0.85 after 30-min and 120-min cold exposure, respectively, but 0.59 at 27°C and 0.77 after 5-min hand immersion. As summarized in Table 5, the cut-off value of Δtemp and accuracy seemed to reach plateau levels 30 min after cold exposure. When the cut-off value for detecting BAT-positive subject was set as 1.01°C for Δtemp after 30-min cold exposure, the sensitivity, specificity, and diagnostic accuracy were 74.3%, 92.3%, and 79.2%, respectively, which were comparable with those after 120-min cold exposure.

**Table 5:** Accuracy for brown adipose tissue activity during cold exposure.

	Cut-off value,°C	Sensitivity, %	Specificity, %	Accuracy, %	AUC
27°C	0.78	28.6	100	47.9	0.59
19°C 30 min	1.01	74.3	92.3	79.2	0.80*
19°C 60 min	1.03	85.7	84.6	85.4	0.89*
19°C 120 min	0.96	80.0	76.9	79.2	0.85*

*P < 0.05, vs. 27°C

## Discussion

In this study, to investigate the optimal index for assessing BAT thermogenic activity using the IRT method, healthy volunteer subjects were exposed to the cold for 2 h, and the skin temperature of the supraclavicular region close to BAT depots (T_scv_) was compared with the metabolic activity (SUV_max_) assessed by the standard ^18^FDG-PET/CT method. Our results showed that the cold-induced response of Δtemp, reflecting the difference between T_scv_ and a control chest region apart from BAT depots (T_c_), was the most relevant index of SUV_max_.

Human BAT is mainly present in the supraclavicular region, which has been the focus of most studies measuring the temperature response by the IRT method or using wire-less thermistors. In a previous thermistor study, a correlation coefficient of r = 0.52 was found between T_scv_ after cold exposure and SUV_max_ [7], which is similar to our result (*r* = 0.48). However, the T_sk_ value itself may be affected by various factors such as the subcutaneous fat thickness [20,21], SkBF, and possibly other thermogenic tissues. Therefore, to minimize the influence of these factors, we calculated Δtemp as the difference between T_scv_ and T_c_, and found a higher correlation coefficient (*r* = 0.74). In fact, T_scv_ and T_c_ correlated with body fatness (% body fat), mean body temperature, and SkBf, while Δtemp showed no significant correlation with these parameters.

Previous reports have shown that BAT activity could be evaluated by the IRT method using thermoneutral conditioning [9,21] or the 5-min hand immersion test [11,12]. These methods are simple and less invasive, and thus would be useful in clinical settings. However, these methods have not been validated in relation to the BAT activity assessed by ^18^FDG-PET/CT. In our study, under a thermoneutral condition without cold exposure, no correlation of T_scv_ and Δtemp with SUV_max_ was found. In the hand immersion test, however, Δtemp correlated with SUV_max_, but with a lower correlation coefficient (0.43) than found in the cold exposure test (*r* = 0.74). Thus, the hand immersion test may be feasible only for subjects with relatively high BAT activity.

In most previous human studies, BAT was assessed after 2 h or longer cold exposure, regardless of the ^18^FDG-PET/CT or IRT method [1, 9]. In this study, we monitored T_sk_ responses to cold exposure at 10~30-min intervals for 120 min, finding that the response in Δtemp reached a steady level after 30 min and was maintained thereafter. In fact, comparative positive correlations between the IRT parameters and SUV_max_ were observed even at 30 min after cold exposure, including that for Δtemp (*r* = 0.68). Accordingly, the ROC analysis for the data after 30-min cold exposure revealed that the sensitivity, specificity, and diagnostic accuracy were similar after a 120-min cold exposure. Thus, 120-min cold exposure, as applied in the previous studies, is not necessary. Our easier protocol of 30-min cold exposure is sufficient for BAT evaluation by the IRT method.

One of the limitations of this study is that all our participants were young and non-obese males. To confirm the overall feasibility of our IRT method, it should also be tested in other groups, particularly in female and/or obese individuals. They have more subcutaneous fat which is insulating, and may influence Δtemp depending on the mass/thickness of the fat. Moreover, Δtemp may not only be influenced by heat directly transmitted from underlying BAT, but also from blood flow in the carotid and subclavian arteries. Further studies are needed to examine the possible confounding effects of these factors.

In conclusion, Δtemp calculated from IRT after 30-min cold exposure highly correlated with SUV_max_ assessed by ^18^FDG-PET/CT. Thus, the IRT method may be useful as a simple and less-invasive alternative for evaluating BAT, particularly for large-scale screening and longitudinal repeat studies.

## Supporting information

S1 File Data sheet Fig 2. (XLSX)

S2 File Data sheet of Fig 3. (XLSX)
